# Properties of paraffin wax as a bolus material in accelerator-based boron neutron capture therapy

**DOI:** 10.2340/1651-226X.2025.44024

**Published:** 2025-09-28

**Authors:** Jenna Tarvonen, Lauri Wendland, Liisa Porra, Tiina Seppälä, Mikko Tenhunen

**Affiliations:** Comprehensive Cancer Center, Helsinki University Hospital (HUS) and University of Helsinki, Helsinki, Finland

**Keywords:** BNCT, AB-BNCT, bolus, dosimetry, paraffin wax

## Abstract

**Background and purpose:**

Boron neutron capture therapy (BNCT) is targeted radiation therapy enabling cellular-level cancer treatment. With epithermal neutrons, the dose maximum typically occurs ~2 cm deep in tissue, challenging superficial tumor control. As in external beam radiation therapy, surface dose can be increased using a bolus. However, in BNCT, tissue equivalency is complex and strongly dependent on elemental composition. This study examined a paraffin wax bolus’s effect on the epithermal neutron beam in accelerator-based BNCT (AB-BNCT) and evaluated agreement between treatment planning system (TPS) calculations and measurements.

**Materials and methods:**

Beam characterization used the neutron activation method with gold and manganese foils. Due to its high cross-section for thermal neutrons, manganese activation serves as a surrogate for boron dose estimation. Irradiations were conducted in a 3D water tank and in a head-shaped phantom with 5 and 10 mm boluses. Dose calculation utilized the newly commissioned RayStation TPS with a Monte Carlo-based engine built on the GEANT4 toolkit.

**Results:**

Calculated and measured results agree within 5% accuracy in significant dose region (>50% dose). Near the surface and at greater depths, agreement remains within 10%. The bolus shifts the activation depth curve toward the surface by 4–13 mm depending on its thickness. Manganese surface activation increases from 30% without a bolus to ~70% and ~ 90% with 5 and 10 mm boluses, respectively.

**Interpretation:**

Paraffin wax effectively moderates neutron energy, making it a suitable bolus material for AB-BNCT treatments requiring increased surface dose.

## Introduction

As a biologically guided radiotherapy modality, boron neutron capture therapy (BNCT) allows precise tumor targeting based on the cellular uptake of boron. In BNCT, the boron dose formation primarily occurs through the thermal neutron capture reaction of the ^10^B isotope in a pharmaceutical compound that selectively accumulates in tumor cells [1, 2]. In the presence of boron, the use of epithermal neutrons generally results in a dose maximum approximately 2 cm within the tissue, as the beam gradually thermalizes. Superficial tumors may not receive adequate surface dose for tumor control. Similar to conventional radiotherapy, the surface dose can be increased by using a bolus to enhance neutron moderation and increase the dose at the skin level. According to the International Commission on Radiation Units and Measurements (ICRU) guidelines [3], the bolus material should simulate the absorption and scattering properties of the target tissue while also being practical and cost-effective.

An accelerator-based BNCT (AB-BNCT) facility at Helsinki University Hospital has started patient treatments in 2025 [4]. The commissioning process included comprehensive verification and validation testing of the system focusing on preparations for clinical studies. This experimental study describes validation of the RayStation treatment planning system (TPS) in the presence of a bolus. The primary objectives are to compare calculated and measured neutron activation results for Mn–Al and Au–Al foils and Mn wires, and to evaluate the influence of the bolus on the displacement of the maximum dose with respect to the phantom surface.

Paraffin and hydrogel boluses were investigated, with paraffin preferred for its accurate and well-known chemical composition, adaptability and durability [5]. Paraffin has a high hydrogen content, approximately 15% by mass compared to around 10% in healthy tissue, leading to stronger elastic scattering and more efficient moderation of epithermal neutrons. As a result, a paraffin bolus shifts the neutron spectrum toward lower energies, with the degree of thermalization increasing with bolus thickness [6].

There is a lack of studies on the use of boluses in BNCT treatments overall. The suitability of a 3D-printed hydrogel bolus has been investigated at an AB-BNCT facility in Japan [7]. The establishment of AB-BNCT facilities in hospitals will expand the possibilities for BNCT treatments. Therefore, it is important to study bolus materials and ensure the accuracy of TPSs in accounting for bolus interactions.

## Materials and methods

### Equipment

A recommended method for neutron dosimetry, specifically for determining neutron fluence required for boron dose calculations, involves neutron activation analysis. In this technique, thin metallic foils made of materials that interact with neutrons, are irradiated in a neutron field, and the resulting activation is measured using a gamma spectrometer [1, 2]. The method is accurate, and achieves systematic uncertainty of approximately 2–3%, while statistical uncertainties below 1% can be attained with appropriate irradiation and spectrometer readout times.

In this study, diluted Mn–Al and Au–Al activation foils (1 wt % Au or Mn, diameter 12 mm, thickness 0.2 mm) and activation wires (Al–Mn, 2.6 wt % Mn, length 10 mm, diameter 0.76 mm) from Goodfellow Cambridge Ltd. were used in the neutron activation analysis. Gold has a significant cross-section (~98 barns at 0.025 eV) for both thermal and epithermal neutrons, while manganese (~13 barns) is primarily sensitive to thermal neutrons. For comparison, ^10^B has a much higher thermal neutron cross-section (~3990 barns), which is the basis of dose formation in BNCT. Thus, Mn and Au activation provide a practical means to characterize the neutron spectrum and thermal fluence relevant for boron dose estimation [2, 8]. The number of activated atoms is reported per monitor unit (MU) of the fission chamber, which is calibrated for the beam output, based on absolute dosimetry using Mn activation foil signal in a standard phantom geometry. The measurements in this study were conducted using MU-based irradiation time, ensuring comparability between different runs.

The number of activated target atoms per MU, *N_act_*/MU/atom, was determined by measuring foil and wire activation with a high-purity gamma spectrometer (GEM Coaxial P-type PopTop HPGe γ-Ray Detector; ORTEC, Zoetermeer, the Netherlands), accounting for the known isotope decay rate after the desired MU-based irradiation time [9].

PLA plastic was used to manufacture 3D printed (Ultimaker 2+ 3D) special holders for foils and wires as well as square 80 mm × 80 mm molding frames with a height of 5 (±0.5) and 10 (±0.5) millimeters to obtain the desired thickness and shape of the boluses. Paraffin granules (HUS Pharmacy, Finland) were melted in a hot water bath and poured to the frames to harden to the right bolus shape and thickness. To compensate for shrinkage and avoid air gaps, the frames were slightly overfilled, and the surfaces leveled after solidification. Challenges in manufacturing reproducibility were included in the uncertainty analysis.

The bolus impact was studied by performing depth profile measurements in a large water phantom (MP3-PL Hi-Tech 3D water phantom solution for particle therapy treatment machines; PTW, Freiburg, Germany) filled with distilled water. Its dimensions are 640 × 630 × 520 mm³, with a thinned entrance window (250 × 250 × 5 mm³) oriented towards the neutron beam. To model a typical patient treatment, a head-shaped water-filled plastic phantom with a 3.2 mm cellulose acetate butyrate (CAB) wall (Radiosurgery Verification Phantom, The Phantom Laboratory, Salem, NY, USA) was also irradiated with a bolus on its surface.

The neutron source in Helsinki University Hospital is an electrostatic proton accelerator with a rotating lithium target manufactured by Neutron Therapeutics, Inc. (Danvers, MA). The proton beam energy is 2.6 MeV and the operating current is 30 mA. It generates a high epithermal neutron flux, similar to the FiR 1 reactor, designed in compliance with the IAEA criteria for beam quality [2]. Due to the wide emission angle from neutron scattering properties and beam production, the beam can be considered divergent, with its directionality parameter (current-to-flux ratio) defined as *J /* ϕ*_epi_* ≥ 0.7 [2]. A circular, lithiated plastic collimator with a 14 cm aperture was utilized in this study [4, 10].

### Dosimetric measurements in the water tank

**Figure 1:**
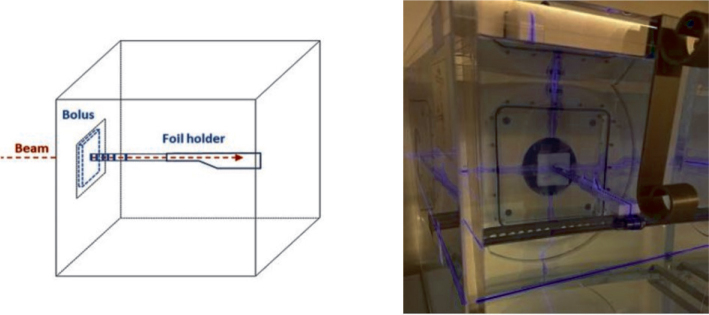
Schematic and snapshot of the experimental setup used for the water tank measurements.

Measurements were carried out in a 3D water tank as shown in Figure 1. Measurement setups were constructed with 5 and 10 mm thick boluses and without the bolus as a reference case.

The bolus was aligned to the hash marks on the tank’s front wall, and the tank was positioned using in-room lasers to center the entrance window along the beam axis and adjusted to ensure submillimeter precision. Aperture-source distance (ASD) and phantom alignment were verified with a spacer tool. Au and Mn foil pairs were placed at defined depths (0–10 cm) in a 3D-printed holder, which was leveled and positioned against the inner tank wall along the beam axis.

The ASD was 2 cm without a bolus and adjusted to 1.5 and 1.0 cm for the 5 and 10 mm boluses, keeping the phantom surface at the same distance from the aperture. This corresponds to the clinical situation, where the source-to-surface distance (SSD) varies depending on bolus use. Beam divergence causes different intensities at each ASD, which was corrected for using the scaling factor *c* in the fitting procedure presented later. Foil positions were unchanged, ensuring measurements at identical depths relative to the surface.

After irradiation, Mn and Au foils were read simultaneously in the spectrometer, with the Au foil placed closer to the Ge crystal to minimize attenuation of the relatively low-energy gammas from activated atoms.

### End-to-end measurements in head-shaped phantom

**Figure 2:**
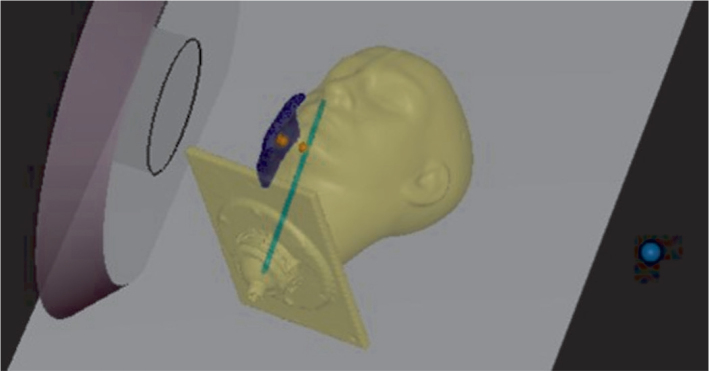
Schematic picture of the measurement setup with the RSVP phantom, demonstrating the arrangement and the alignment of the bolus, the foils and the wire-rod relative to the beam aperture. The foil pairs are marked with orange circles, and the green line represents the rod to which the Mn wires were evenly placed within the area covered by the bolus.

The study with the RSVP (Radiosurgery Verification Phantom) head phantom was carried out according to the actual treatment planning protocol. CT imaging for dose calculation and image-guided positioning was performed using an in-room, rail-mounted CT scanner (Somatom Confidence, Siemens Healthineers). Image-guided positioning was performed with a six-axis robotic system, Exacure, manufactured by BEC GmbH (Reutlingen, Germany), to accurately set and align the phantom to the planned treatment position. The water-filled phantom was positioned on the patient base plate with specialized head support and tilted so that the beam axis was directed towards the ‘parotid’ region, where an anatomically shaped 10 mm thick bolus was placed. Three pairs (Mn & Au) of foils were positioned along the beam axis at the entry point under the bolus, at the inner surface of the phantom and at the center of a horizontally placed wire rod, approximately 2 cm deep within the phantom as shown in the schematic picture in Figure 2. Mn wires were evenly positioned along the rod, allowing for an analysis of the agreement between calculated and measured values across the treatment area, perpendicular to the beam axis. The ASD was set to be 5 cm in RSVP phantom measurements.

### Analysis

Calculation of the activated target atoms was done using a GEANT4 [11]- based Monte Carlo dose-engine interfaced through the RayStation (RaySearch Laboratories, Stockholm, Sweden, 2023B) TPS. The bolus material was set to ‘wax’ which mass fraction matched with paraffine wax, and default thickness was used. In the water tank simulations, a voxel size of 5 × 5 × 1 mm³ was used for the calculations, whereas for RSVP phantom, a voxel size of 2 × 2 × 2 mm³, which is in use in clinical treatment plans, was employed. The number of iterations was set to 5 in both phantom setups from which the statistical uncertainty was calculated as the standard deviation of five runs divided by the average value.

All the activation foils were read at the basic nominal position with the automated sample changer. The Mn wires were manually exchanged and read closer to the Ge crystal, which shortened the reading time. Therefore, the results obtained proximally must be multiplied by a correction factor *k_prox→nom._* = 6.98 ± 0.08, which was determined through repetition experiments by reading the same wire or foil in both readout geometries.

Activation and readout response of the foils and wires differ due to the geometric differences and Au/Mn content. This is accounted for by multiplying the Mn wire results by a conversion factor *k_wire→foil_* = 1.088 ± 0.013, derived from repeated measurements in the large water tank with wires and foils placed at 2 cm depth in the same setup.

After the previously stated corrections and conversions, the ratio *N*_act_(calculated)/*N*_act_(measured) was calculated. All the results in this work were analyzed and visualized using the ROOT software [12].

To investigate the effect of bolus thickness on the positioning of the neutron activation depth profile relative to the phantom surface, a fit using the ROOT-based Minuit algorithm [13] on data obtained from different measurement setups was performed. The fitting process determines the values of two variables, *∆* and *c*, by minimizing Equation 1


$$f_{2}(x)\;=\;c\;x\;f_{1}(x)(x\;+\;\Delta )$$

where *∆* is the shift between the intensity maxima of the two curves, and *c* is a scaling factor in the amplitude direction, meaning, it scales differences in activation intensity.

*f*_1_ represents the reference depth dose (activation) distribution data without a bolus, while *f*_2_ is the bolus data which are being compared to *f*_1_.

## Results

### Dosimetric measurements in the water tank

**Figure 3:**
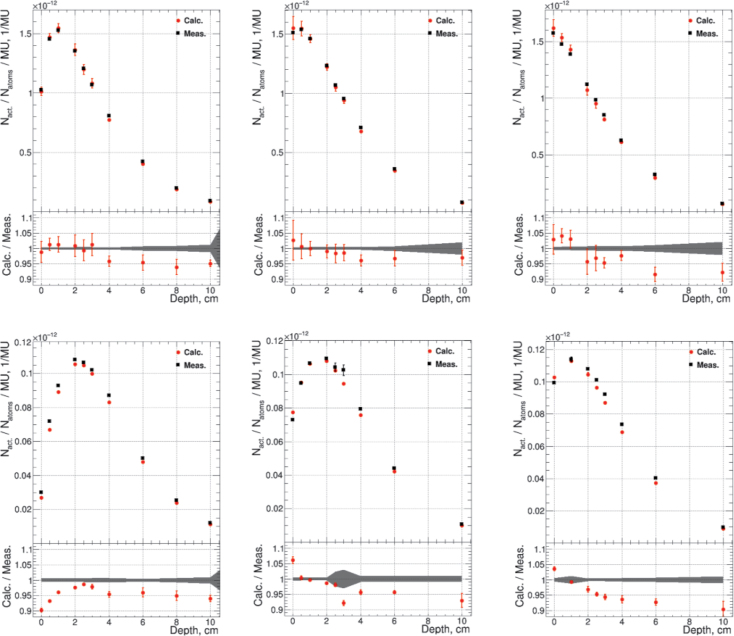
Combined graphs showing an overview of depth profiles and ratio plots. The upper part of each figure shows the number of activated target atoms/MU for Au- (top row) and Mn (bottom row) foils as a function of depth. The lower part presents the calculated-to-measured ratio for no bolus (left), 5 mm bolus (middle), and 10 mm bolus (right). Uncertainties represent statistical uncertainties, where the gray blocks in ratio are uncertainties for measured and the red error bars for calculated values accordingly.

The results obtained from the water tank measurements are shown in Figure 3, which presents the calculated and measured number of activated target atoms of Mn and Au foils per MU as a function of depth. The lower part of the figure displays the ratio *N_act_*(calculated)/*N_act_*(measured), across the depth range.

In measurements without a bolus, the significant dose region (where activation exceeds 50% of the maximum) extends from ~0.5 to 5 cm in depth. Calculated and measured values match within 5% (1–4% total uncertainty, 1σ) in this region. Across the entire measurement range, from the surface to a depth of 10 cm, the agreement remains within 10% (1–4% total uncertainty, except at the surface point ~ 10%, 1σ).

With the bolus, the peak neutron activation intensity shifts toward the surface for both materials. However, particularly for gold, the intensity decreases more rapidly below 50% of the maximum, slightly before a 4 cm depth. Nevertheless, also with boli, the calculated and measured values agreed within 5% (total uncertainty 1–3%, 1σ; slightly higher at the surface) in the clinically relevant dose region, and within 10% (total uncertainty 2–4%, 1σ) across the full measurement range, remaining within the biological uncertainties related to the BNCT procedure.

Overall, the calculation tends to slightly underestimate the neutron activation, except at the surface when the bolus is used. This is especially seen with manganese where the calculated activation values are 3–6% higher compared to the measured ones. Additionally, when bolus is used, a decreasing linear trend is observed in the ratio *N_act_*(calculated)/*N_act_*(measured), suggesting that the depth coordinate has a small offset between the calculation and the measurement.

### End-to-end measurements in head-shaped phantom

**Figure 4:**
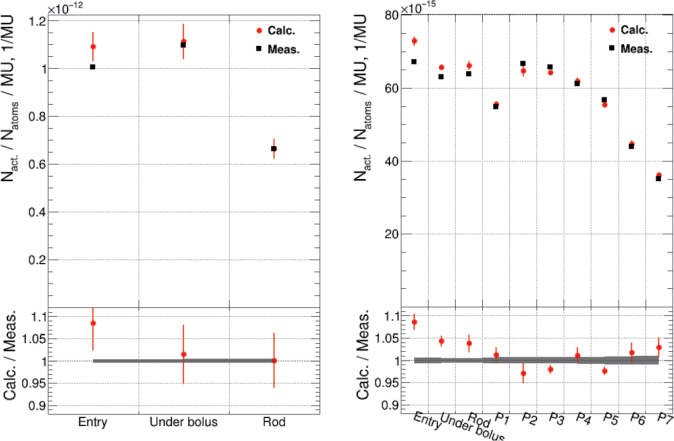
Neutron activation and the calculated-to-measured ratio inside the RSVP phantom at the points ‘entry’, ‘under the bolus’ and ‘rod’ for Au foils (left). Activation of Mn foils and Mn wires at horizontal positions P1 (superior) to P7 (inferior) inside the phantom is shown in the right-side image. Uncertainties represent statistical uncertainties, where the gray blocks in the ratio plot indicate the uncertainties for measured values, and the red error bars correspond to the uncertainties for calculated values.

The results obtained from the RSVP head phantom measurements are presented in Figure 4. The calculated Mn wire activation values (P1–P7) agree with the measured ones within 3% (total uncertainty of 2–3%, 1σ). For the foil pairs the agreement depends on the isotope. The calculated values for gold align with the measurements, within 2%, with a total uncertainty of 6.4–6.7% (1σ). For manganese, the agreement is within 4%, with a lower total uncertainty of 2% (1σ).

The correlation is slightly weaker, 9% (total uncertainty of 2.1% for Mn and 6.4% for Au, 1σ), only at the entry point, but this is likely due to greater uncertainties caused by the surface geometry and material interface.

### Shift of the maximum dose

Measured and calculated results showed increased manganese activation at the phantom surface in the presence of a bolus. Without a bolus, the surface dose is approximately 30% of the maximum, increasing to ~70% with a 5 mm bolus and to ~ 90% with a 10 mm bolus.

To assess the effect of bolus thickness on the location of maximum dose, depth dose curves between the no-bolus (0 mm) case and with 5 and 10 mm boli were fitted and the shift was calculated. Additionally, measured and calculated results were compared separately to avoid any additional sources of uncertainty. Due to increased uncertainty from a less homogeneous energy spectrum near the surface and at greater depths, the fitting was limited to the significant dose region between 0.5 and 5 cm depths.

The fitting results from the measured data show that the 5 mm paraffin bolus shifts the depth curve toward the surface by approximately 4.3 ± 0.4 mm for Mn and 3.9 ± 1.2 mm for Au. With the 10 mm bolus, the corresponding shifts are 9.0 ± 1.5 and 7.0 ± 3.6 mm, respectively, indicating a smaller effective bolus thickness than expected. In contrast, the fits to the calculated data yield shifts of 7.6 ± 0.1 mm (Mn) and 7.8 ± 0.2 mm (Au) for the 5 mm bolus, and 13.3 ± 0.1 mm (Mn) and 13.4 ± 0.2 mm (Au) for the 10 mm bolus. These calculated shifts are closer to the ideal values of 6.2 and 12.4 mm, determined based on the theoretical composition of paraffin wax (C₂₅H₅₂).

**Figure 5:**
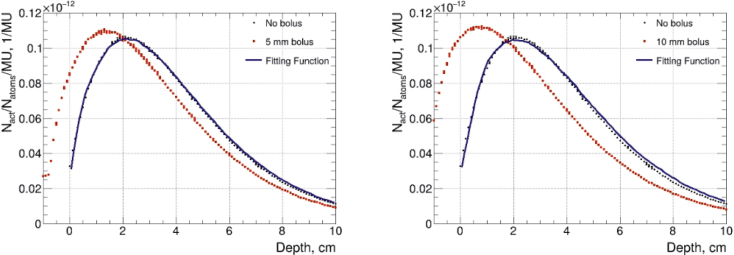
Fitting curve (continuous curve), data without bolus (circles) and with bolus (squares) from calculations with 5 mm (left) and with 10 mm (right) paraffin wax bolus using Mn- activation foils. Only statistical uncertainties included.

Figure 5 presents the shift of depth dose curves measured with Mn activation foils. It shows the reference data obtained from calculations without the bolus, along with fitted graphs derived by comparing the data calculated with the 5 and 10 mm boluses to the reference data. A calculation resolution of 1 mm was used.

### The effect of the bolus material density

**Figure 6:**
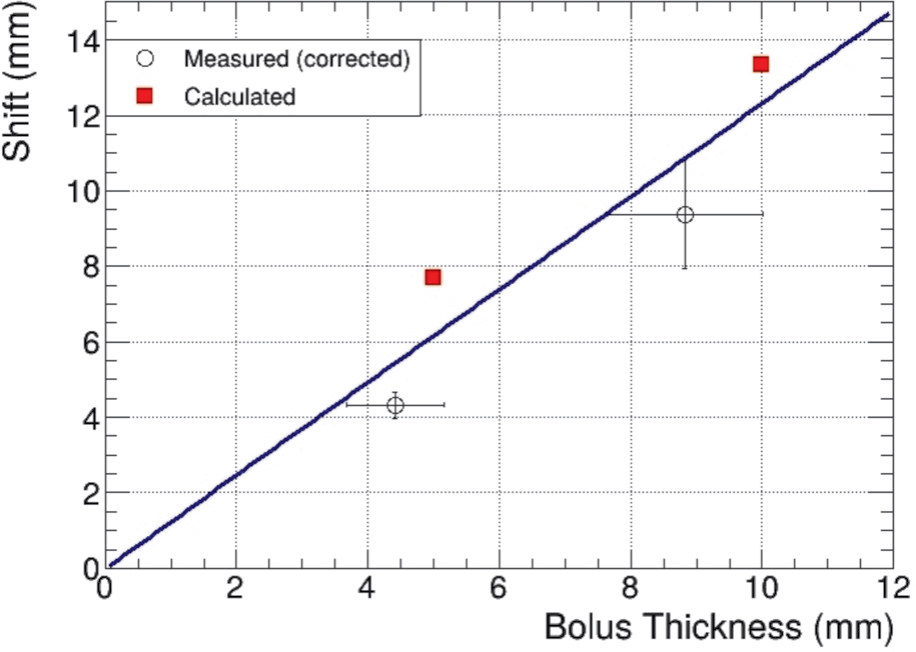
Measured and calculated shift- values with uncertainties as a function of bolus thickness. The uncertainty bars for the measured points depict the combined uncertainty arising from the density determination method and the bolus manufacturing process.

The decreasing linear trend in the calculated-to-measured ratio, along with the lower-than-expected effective thickness, suggests that the bolus density may be less than the literature value [14] used in the TPS. Therefore, the bolus density was determined by weighing appropriately sized pieces and measuring the displaced water volume in a graduated cylinder. The resulting density, 0.82 ± 0.09 g/cm^3^, was lower than the value, 0.93 g/cm^3^ used in the calculations. Figure 6 presents a linear fit of the measured and calculated shifts with uncertainties as a function of bolus thickness, where the measured thickness values have been corrected by the density ratio of 0.82/0.93. The corrected bolus thicknesses are 4.4 ± 0.7 mm and 8.8 ± 1.2 mm. The total uncertainty includes the density determination method and the variation in bolus thickness resulting from the manufacturing process.

The fitting allows for accounting the effect of density on the magnitude of the shift. The predicted shifts using the fit are 6.2 mm for the 5 mm bolus and 12.3 mm for the 10 mm bolus, which are very close to the ideal values.

### Uncertainties

In Monte Carlo-based dose calculations, statistical uncertainties arise from finite sampling and decrease with the square root of the sample size. In RayStation, the TPS dose engine estimates this by calculating the average relative statistical uncertainty within a selected isodose surface. In this study, the statistical uncertainty of the TPS was defined as the standard deviation from five separate calculation runs.

In the activation foil measurements, statistical uncertainty is mainly due to counting statistics in gamma spectrometry, governed by the stochastic nature of radioactive decay. Higher count rates and longer acquisition times reduce the uncertainty.

Regarding systematic uncertainties, the calibration of the spectrometer is the most significant one; however, it cancels out in the relative calculations. Therefore, the dominant systematic uncertainty source in the measurements is the manual alignment of the water tank, estimated at 1% of the ASD. The uncertainty in foil placement in depth direction was 0.2 mm, corresponding to 0.2–1.5%, but was negligible when a bolus was used. Bolus thickness variation, measured to be ± 0.5 mm, led to 0.3–1.0% uncertainty depending on the depth. The statistical uncertainty from measurements was combined with the systematic uncertainty using error propagation. The total uncertainty for the ratio of the calculated to the measured result was obtained by quadratically combining the statistical uncertainty of the calculation with the total uncertainty of the measurement.

The density of the paraffin bolus was determined via Archimedes’ principle. The main source of uncertainty was the reading accuracy of the 50 mL graduated cylinder (± 1.25 mL), resulting in an 11.4% uncertainty in volume. This was quadratically combined with the thickness variation of the bolus to yield the total uncertainty of the density.

Table 1 summarizes all relevant uncertainties.

**Table 1:** Uncertainties and their magnitudes.

Uncertainty	Calculated	Measured
Statistical	0.1–9%	1–4%
ASD		1.0%
Bolus		0.3–1.0%
Detector placement in depth direction		0.2–1.5% without a bolus, negligible with bolus
*k_prox→nom_*(Mn wires only)		1.0%
*k_wire→foil_* (Mn wires only)		0.13%
Density		
Scale		0.0001/8.9901 g causing 0.0011% uncertainty
Cylinder		1.25/11.00 ml causing 11.4 % uncertainty

ASD: Aperture-source distance.

## Discussion and conclusion

Paraffin wax is a feasible bolus material for AB-BNCT. TPS calculations agreed with measurements within 5% in the dose-relevant region. Manganese activation at the phantom surface was observed to increase from 30% to 70% and to 90% of the maximum, depending on the bolus thickness. Measured shifts in maximum dose showed slightly reduced effective bolus thicknesses due to lower material density, which can be corrected by measuring true bolus density and applying the presented prediction model.

Calculating radiation doses with neutrons is challenging due to their interactions with matter being highly dependent on energy. This increases the uncertainty of simulations, particularly at material interfaces, where sharp transitions can amplify inaccuracies.

Each voxel can represent only one material selected from a pre-defined list of supported materials, potentially causing minor inaccuracies. Tests with smaller voxel sizes and adjusted material–air interfaces showed negligible effects on results, indicating that with careful voxel boundary definition, partial volume effects are minimal.

The TPS generally slightly underestimated activation, except at the surface when a bolus is used. This surface discrepancy may partly result from air gaps between the bolus and phantom, but, more significantly, from the bolus having a lower-than-expected density due to casting inhomogeneities and air bubbles. The heating and cooling involved in manufacturing may alter the microstructure and phase composition of paraffin wax, leading to permanent changes in density and hydrogen content, which affect its neutron moderation capability [15]. The reduced hydrogen content diminishes neutron moderation, resulting in smaller effective bolus thicknesses.

However, no significant reduction in effective thickness was observed with the RSVP phantom, likely due to improved bolus contact on the curved surface, reducing air gaps and minimizing partial volume effects. The smaller voxel size used in simulations may also enhance accuracy. Nevertheless, density-related variation in effective thickness must be considered in treatment planning, as it may cause the boron dose maximum to shift toward the skin less than intended. In clinical practice, the uncertainty caused by bolus density is relatively small compared to other sources, but, if necessary, the actual measured density can be used by manually setting the value in the TPS to scale the dose calculation accordingly. The prediction curve presented in Figure 6 provides a reliable means to correct for the density effect.

In conclusion, this study confirms reliable surface dose estimation and supports the clinical use of paraffin boluses in superficial treatments with AB-BNCT at Helsinki University Hospital. Paraffin was chosen over hydrogel [5] for its higher hydrogen content, stronger neutron moderation, low cost, durability and ease of shaping, making it particularly suitable as a bolus material for enhancing neutron moderation and surface dose build-up. Consistent clinical use can be ensured through QA measures such as density verification when required.

## Data Availability

The data are available from the corresponding author upon reasonable request.
